# Price and Prejudice? The Value of Chimeric Antigen Receptor (CAR) T-Cell Therapy

**DOI:** 10.3390/ijerph191912366

**Published:** 2022-09-28

**Authors:** Gyeyoung Choi, Gyeongseon Shin, SeungJin Bae

**Affiliations:** College of Pharmacy, Ewha Womans University, Seoul 03760, Korea

**Keywords:** chimeric antigen receptor T-cell therapy, CAR T-cell, economic evaluation, value

## Abstract

Although chimeric antigen receptor (CAR) T-cell therapy has shown a high response rate in lymphoma patients, its cost-effectiveness is controversial due to the high price and uncertainty of the clinical evidence. In addition to the high acquisition cost of CAR T-cell therapy, procedure and facility cost increase the financial burden considering the frequency of adverse events such as cytokine release syndrome. In clinical research, relatively short follow-up periods were used compared to traditional cancer agents. In addition, head-to-head comparative effectiveness data are unavailable, which is an important factor when evaluating the cost-effectiveness of a new treatment. Additional evidence that will compensate for the uncertainty of existing clinical data is needed for full evaluation of long-term efficacy, safety, and comparative effectiveness.

## 1. Introduction

Since tisagenlecleucel (Kymriah^®^) was approved by the Food and Drug Administration (FDA) in 2017 as the first-in-class chimeric antigen receptor (CAR) T-cell therapy for the treatment of relapsed/refractory acute lymphoblastic leukemia (ALL), four additional CAR T-cell therapy products have been approved [1]. Due to its groundbreaking treatment mechanism and high response rate in clinical trials [2], CAR T-cell therapy is regarded as the most promising treatment for intractable hematologic malignancies [3]. We searched CAR T-cell therapy clinical trials through PubMed and Google Scholar, with the population being mainly adult patients with lymphoma, interventions being CAR T-cell therapy approved by the FDA, comparisons being the standard of care for the specific lymphoma, and outcomes being objective response rate, complete response rate, and adverse events. The search terms included tisagenlecleucel, axicabtagene ciloleucel, brexucabtagene autoleucel, lisocabtagene maraleucel, idecabtagene vicleucel, and ciltacabtagene autoleucel. The search was performed between 1 August 2022 to 15 September 2022. As a result, twelve studies for the six CAR T-cell therapies were selected. Table 1 summarizes the CAR T-cell therapies and the information for their representative clinical trials. In clinical trials of tisagenlecleucel [4], complete remission occurred in 6 of 14 patients (43%) with diffuse large B-cell lymphoma (DLBCL) and 10 of 14 patients (71%) with follicular lymphoma. All patients in complete remission by 6 months remained in remission 7.7 to 37.9 months (median, 29.3 months) after induction [4]. In another clinical study, 89 of 108 assessable patients (82%) with refractory large B-cell lymphoma treated with axicabtagene ciloleucel achieved an objective response, and complete responses were noted in 63 patients (58%) [5]. This is a marked improvement compared with salvage chemotherapy regimens including rituximab in large B-cell lymphoma patients, where 20% survived at 5 years of follow-up, and only 7% achieved a complete response [6]. CAR T-cell therapy provides a promising option for patients with hematologic malignant neoplasm where standard treatment does not respond or exist, and may cure patients with ALL without transplant [7,8,9,10].

Despite the remarkable outcomes in clinical trials, there are barriers to access CAR T-cell therapies. Obviously, high price is the first barrier. The acquisition cost of CAR T-cell is between $373,000 to $475,000 per infusion, excluding extra procedures and facility costs [23]. Moreover, the therapies are usually performed in an inpatient environment since it requires the infusion of modified T cells and consequent monitoring of the disease status, costing an additional $79,466 to $85,267 [10].

The second barrier is the serious adverse effects. The development of cytokine release syndrome was the most frequent consequence of CAR T-cell immunotherapy (CRS) [24]. In the phase 1 and 2 clinical study of tisagenlecleucel, CRS occurred in 58 of 75 patients (77%) and 35 of 75 patients (47%) were admitted to the intensive care unit for management of CRS [11]. CRS is a systemic inflammatory response observed after adoptive T-cell therapy [25]. It is triggered by activated T-cells releasing cytokines and chemokines, as do other immune cells such as monocytes, macrophages, and dendritic cells [26]. Severe increases in cytokine levels can affect any organ in the body, including cardiovascular, respiratory, integumentary, gastrointestinal, hepatic, renal, hematological, and nervous systems, and can be lethal to cancer patients [27]. The cost of treating CRS ranges from $30,000 to $56,000 per patient [28]. The total treatment cost for CAR T-cell therapy has been estimated to reach up to $500,000 for patients with severe CRS [28].

The last barrier is the lack of clinical data available for cost-effectiveness analysis. To conduct cost-effectiveness analysis, we need to know if the improved survival/response rate observed in clinical trials is attributable to the difference in patient characteristics or treatment regimens [23,29,30,31,32]. However, CAR T therapy has been approved based on single-arm, open-label, phase 1 or 2 studies [4,11,30], which leads to a weakness of understanding of the comparative effectiveness of CAR T-cell therapy vs. comparator(s). Several studies have assessed the value of CAR T-cell therapy and reported a favorable cost-effectiveness ratio when the willingness to pay threshold is $150,000/QALY [23,29,31,33], but there are many uncertainties. Furthermore, CAR T-cell therapies lack long-term effectiveness data, which are a critical determinant of cost-effectiveness. As summarized in Table 1, the longest median follow-up period of a CAR T-cell therapy was 40.3 months of tisagenlecleucel. In most other studies the median follow-up period was roughly around 12 months. In a study conducted by Lin et al. (2018) [29], the authors relied on the assumption of a 40% 5 year relapse-free survival rate that resulted in a 21.1 year increase in life expectancy and $61,000 per life-year or quality-adjusted life year (QALY) gained. However, there is the possibility of a 20% or even lower 5 year relapse-free survival rate, and this would reduce QALYs and increase costs.

In 2021, 299 new agents were added to the pipeline for CAR T-cell therapy, which is a 35% increase from 2020 [34]. Various treatment options that might provide improved efficacy and safety are being developed. However, from the payers’ perspective, an increasing number of expensive therapies with uncertain clinical value could raise affordability issues, given that the healthcare budget is limited in public health insurance.

## 2. Proposal & Conclusions

Due to the high price of CAR T-cell therapy, cost-effectiveness analysis plays a significant role in evaluating the value of the drug and providing treatment options. We make several suggestions to address the uncertainty raised earlier in the cost-effectiveness analysis of the therapy.

### 2.1. Long-Term Follow-Up Studies with a Larger Number of Participants Are Needed

The need for long-term follow-up, comparative effectiveness, and safety data has been repeatedly brought up as a way to reduce uncertainty in the economic evaluation of CAR T-cell therapy [23,30,32]. As mentioned above, the value assessments were mainly based on phase 1 or 2, single-arm studies that were performed in a small number of subjects for a relatively short period. Because long-term follow-up data are not available, whether CAR T-cell therapy is safe and effective compared to existing options remains unclear from a long-term perspective. 

In addition, although the indications for CAR T-cell therapies are expanding through clinical studies, a limited number of patients were included in each trial of lymphoma. The cost-effectiveness study of Sakar et al. [30] used the results of a phase 2 trial of pediatric relapsed/refractory B-cell acute lymphoblastic leukemia, which showed an overall remission rate of 81% among 75 patients [11]. However, there was a fraction of patients who could not receive the intended CAR T therapy due to manufacturing issues or death and the outcomes of these patients were not reported. Incorporating these patients into the model would further increase the uncertainty of cost-effectiveness [30]. Obtaining additional data is not free, yet it may be the most fundamental solution.

### 2.2. Head-to-Head Studies Should Be Conducted

Adding the results of head-to-head trials could also lower the uncertainty of cost-effectiveness analysis. The CAR T-cell therapy outcomes used in existing analyses were compared with historical controls and comparative evidence is lacking. There are phase 3 studies that compared CAR T-cell therapy with standard of care therapy, although only preliminary results are available (BELINDA study [35] and ZUMA-7 study [36]). Incorporating direct comparison data into the models would increase the reliability of cost-effectiveness analysis.

### 2.3. Efforts beyond Valuation

In the meantime, an outcome-based risk sharing agreement, which reimburses patients based on outcome (i.e., response rate) and collects patient-level data, may be an option to decrease uncertainty from a payer’s perspective. In addition, pharmaceutical companies should also make an effort to lower the price by increasing the transparency of the pharmaceutical supply chain or changing the manufacturing process of CAR T-cells to in-house manufacturing under current good manufacturing practice (cGMP) conditions.

The price of CAR T-cell therapy is obviously high. The prejudice related to its value can only be addressed by collecting relevant data and reducing the uncertainty of cost-effectiveness analysis.

## Figures and Tables

**Table 1:** CAR T-cell therapies and their clinical trial information.

CAR T-Cell Therapy	Population	Study Phase (*n*)	Study Design	Median Follow-Up Period	Efficacy	Safety	Ref.
tisagenlecleucel	Pediatric and young adult patients with B-cell precursor acute lymphoblastic lymphoma that is refractory or in second or later relapse	2 (*n* = 75)	Open-label, single arm	13.1 months	ORR 81% (95% CI, 71 to 89), CRR 60%	CRS 77%	[11]
	Adult patients with relapsed or refractory (r/r) large B-cell lymphoma after two or more lines of systemic therapy	2 (*n* = 93)	Open-label, single arm	40.3 months	ORR 53.0% (95% CI 43.5–62.4), CRR 39%	CRS 27%	[12]
	Adult patients with relapsed or refractory (r/r) follicular lymphoma (FL) after two or more lines of systemic therapy	2 (*n* = 94)	Open-label, single arm	16.59 months	CRR 69.1% (95% CI, 58.8–78.3), ORR 86.2% (95% CI, 77.5–92.4)	CRS 48.5% (grade ≥ 3, 0%), neurological events 37.1% (grade ≥ 3, 3%) ICANS 4.1% (grade ≥ 3, 1%)	[13]
axicabtagene ciloleucel	Adult patients with relapsed or refractory large B-cell lymphoma after two or more lines of systemic therapy	2 (*n* = 92)	Open-label, single arm	15.4 months	ORR 82% (95% CI, 72–89), CR 54%	CRS grade ≥ 3, 13%	[14]
	Adult patients with relapsed or refractory follicular lymphoma after two or more lines of systemic therapy	2 (*n* = 40)	Open-label, single arm	15.9 months	ORR 89% (95% CI, 75–97) CRR 78% (95% CI, 62–90)	CRS grade ≥ 3, 8%	[15]
	Adult patients with large B-cell lymphoma that is refractory to first-line chemoimmunotherapy or that relapses within 12 months of first-line chemoimmunotherapy	3 (*n* = 359)	Randomized	24.9 months	ORR: 83% vs. 50% SOC, odds ratio: 5.31 [95% CI: 3.1–8.9; *p* < 0.0001], CRR 65% vs. 32% SOC	CRS grade ≥ 3, 6%; neurologic events grade ≥ 3, 21%	[16]
brexucabtagene autoleucel	Adult patients with relapsed/refractory mantle cell lymphoma	2 (*n* = 60)	Open-label, single arm	12.3 months	ORR 93% (95% CI, 84 to 98) CRR 67% (95% CI, 53 to 78)	CRS grade ≥ 3, 15%; neurologic events grade ≥ 3, 31%	[17]
	Adult patients with relapsed or refractory (r/r) B-cell precursor acute lymphoblastic leukemia	2 (*n* = 55)	Open-label, single arm	16.4 months	CRR 56%	CRS grade ≥ 3, 24%; neurological events grade ≥ 3, 25%	[18]
lisocabtagene maraleucel	Adult patients with relapsed or refractory large B-cell lymphoma after two or more lines of systemic therapy	1 (*n* = 269)	Open-label, single arm	18.8 months	ORR 73%, (95% CI 66.8–78.0), CRR 53%, (95% CI 46.8–59.4)	CRS 42% (grade ≥ 3, 2%), neurological events 30% (grade ≥ 3, 10%)	[19]
	Adult patients with diffuse large B-cell lymphoma (DLBCL) not otherwise specified (including DLBCL arising from indolent lymphoma), high-grade B-cell lymphoma, primary mediastinal large B-cell lymphoma, and follicular lymphoma grade 3B	3 (*n* = 184)	Randomized	6.2 months	CRR 66% vs. 39% SOC	CRS 49% (grade ≥ 3, 1%), neurological events 12% (grade ≥ 3, 4%)	[20]
idecabtagene vicleucel	Adult patients with relapsed or refractory multiple myeloma after four or more prior lines of therapy	2 (*n* = 128)	Open-label, single arm	13.3 months	ORR 73% CRR 33%	CRS 84% (grade ≥ 3, 5%)	[21]
ciltacabtagene autoleucel	Adult patients with relapsed or refractory multiple myeloma after four or more prior lines of therapy	1b/2 (*n* = 97)	Open-label, single arm	12.4 months	ORR 97% (95% CI 91.2–99.4), CRR 67%	CRS 95% (grade ≥ 3, 4%)	[22]

CI, confidence interval; CRR, complete response rate; CRS, cytokine release syndrome; ORR, objective response rate; SOC, standard of care.

## Data Availability

Not applicable.

## References

[1] Borgert R. (2021). Improving Outcomes and Mitigating Costs Associated with CAR T-Cell Therapy. Am. J. Manag. Care.

[2] Rafiq S., Hackett C.S., Brentjens R.J. (2020). Engineering strategies to overcome the current roadblocks in CAR T cell therapy. Nat. Rev. Clin. Oncol..

[3] Xu D., Jin G., Chai D., Zhou X., Gu W., Chong Y., Song J., Zheng J. (2018). The development of CAR design for tumor CAR-T cell therapy. Oncotarget.

[4] Schuster S.J., Svoboda J., Chong E.A., Nasta S.D., Mato A.R., Anak O., Brogdon J.L., Pruteanu-Malinici I., Bhoj V., Landsburg D. (2017). Chimeric Antigen Receptor T Cells in Refractory B-Cell Lymphomas. N. Engl. J. Med..

[5] Locke F.L., Ghobadi A., Jacobson C.A., Miklos D.B., Lekakis L.J., Oluwole O.O., Lin Y., Braunschweig I., Hill B.T., Timmerman J.M. (2019). Long-term safety and activity of axicabtagene ciloleucel in refractory large B-cell lymphoma (ZUMA-1): A single-arm, multicentre, phase 1–2 trial. Lancet Oncol..

[6] Roth J.A., Sullivan S.D., Lin V.W., Bansal A., Purdum A.G., Navale L., Cheng P., Ramsey S.D. (2018). Cost-effectiveness of axicabtagene ciloleucel for adult patients with relapsed or refractory large B-cell lymphoma in the United States. J. Med. Econ..

[7] Brudno J.N., Kochenderfer J.N. (2018). Chimeric antigen receptor T-cell therapies for lymphoma. Nat. Rev. Clin. Oncol..

[8] Dotti G., Gottschalk S., Savoldo B., Brenner M.K. (2014). Design and development of therapies using chimeric antigen receptor-expressing T cells. Immunol. Rev..

[9] Hartmann J., Schussler-Lenz M., Bondanza A., Buchholz C.J. (2017). Clinical development of CAR T cells-challenges and opportunities in translating innovative treatment concepts. EMBO Mol. Med..

[10] Lyman G.H., Nguyen A., Snyder S., Gitlin M., Chung K.C. (2020). Economic Evaluation of Chimeric Antigen Receptor T-Cell Therapy by Site of Care Among Patients With Relapsed or Refractory Large B-Cell Lymphoma. JAMA Netw. Open.

[11] Maude S.L., Laetsch T.W., Buechner J., Rives S., Boyer M., Bittencourt H., Bader P., Verneris M.R., Stefanski H.E., Myers G.D. (2018). Tisagenlecleucel in Children and Young Adults with B-Cell Lymphoblastic Leukemia. N. Engl. J. Med..

[12] Schuster S.J., Tam C.S., Borchmann P., Worel N., McGuirk J.P., Holte H., Waller E.K., Jaglowski S., Bishop M.R., Damon L.E. (2021). Long-term clinical outcomes of tisagenlecleucel in patients with relapsed or refractory aggressive B-cell lymphomas (JULIET): A multicentre, open-label, single-arm, phase 2 study. Lancet Oncol..

[13] Fowler N.H., Dickinson M., Dreyling M., Martinez-Lopez J., Kolstad A., Butler J., Ghosh M., Popplewell L., Chavez J.C., Bachy E. (2022). Tisagenlecleucel in adult relapsed or refractory follicular lymphoma: The phase 2 ELARA trial. Nat. Med..

[14] Neelapu S.S., Locke F.L., Bartlett N.L., Lekakis L.J., Miklos D.B., Jacobson C.A., Braunschweig I., Oluwole O.O., Siddiqi T., Lin Y. (2017). Axicabtagene ciloleucel CAR T-cell therapy in refractory large B-cell lymphoma. N. Engl. J. Med..

[15] Neelapu S.S., Dickinson M., Munoz J., Ulrickson M.L., Thieblemont C., Oluwole O.O., Herrera A.F., Ujjani C.S., Lin Y., Riedell P.A. (2022). Axicabtagene ciloleucel as first-line therapy in high-risk large B-cell lymphoma: The phase 2 ZUMA-12 trial. Nat. Med..

[16] Locke F.L., Miklos D.B., Jacobson C., Perales M.-A., Kersten M.J., Oluwole O.O., Ghobadi A., Rapoport A.P., McGuirk J.P., Pagel J.M. (2021). Primary Analysis of ZUMA-7: A Phase 3 Randomized Trial of Axicabtagene Ciloleucel (Axi-Cel) Versus Standard-of-Care Therapy in Patients with Relapsed/Refractory Large B-Cell Lymphoma. Blood.

[17] Wang M., Munoz J., Goy A., Locke F.L., Jacobson C.A., Hill B.T., Timmerman J.M., Holmes H., Jaglowski S., Flinn I.W. (2020). KTE-X19 CAR T-cell therapy in relapsed or refractory mantle-cell lymphoma. N. Engl. J. Med..

[18] Shah B.D., Ghobadi A., Oluwole O.O., Logan A.C., Boissel N., Cassaday R.D., Leguay T., Bishop M.R., Topp M.S., Tzachanis D. (2021). KTE-X19 for relapsed or refractory adult B-cell acute lymphoblastic leukaemia: Phase 2 results of the single-arm, open-label, multicentre ZUMA-3 study. Lancet.

[19] Abramson J.S., Palomba M.L., Gordon L.I., Lunning M.A., Wang M., Arnason J., Mehta A., Purev E., Maloney D.G., Andreadis C. (2020). Lisocabtagene maraleucel for patients with relapsed or refractory large B-cell lymphomas (TRANSCEND NHL 001): A multicentre seamless design study. Lancet.

[20] Kamdar M., Solomon S.R., Arnason J.E., Johnston P.B., Glass B., Bachanova V., Ibrahimi S., Mielke S., Mutsaers P.G., Hernandez-Ilizaliturri F.J. (2021). Lisocabtagene maraleucel (liso-cel), a CD19-directed chimeric antigen receptor (CAR) T cell therapy, versus standard of care (SOC) with salvage chemotherapy (CT) followed by autologous stem cell transplantation (ASCT) as second-line (2L) treatment in patients (Pts) with relapsed or refractory (R/R) large B-cell lymphoma (LBCL): Results from the randomized phase 3 transform study. Blood.

[21] Munshi N.C., Anderson L.D., Shah N., Madduri D., Berdeja J., Lonial S., Raje N., Lin Y., Siegel D., Oriol A. (2021). Idecabtagene vicleucel in relapsed and refractory multiple myeloma. N. Engl. J. Med..

[22] Berdeja J.G., Madduri D., Usmani S.Z., Jakubowiak A., Agha M., Cohen A.D., Stewart A.K., Hari P., Htut M., Lesokhin A. (2021). Ciltacabtagene autoleucel, a B-cell maturation antigen-directed chimeric antigen receptor T-cell therapy in patients with relapsed or refractory multiple myeloma (CARTITUDE-1): A phase 1b/2 open-label study. Lancet.

[23] Fiorenza S., Ritchie D.S., Ramsey S.D., Turtle C.J., Roth J.A. (2020). Value and affordability of CAR T-cell therapy in the United States. Bone Marrow Transpl..

[24] Lei W., Xie M., Jiang Q., Xu N., Li P., Liang A., Young K.H., Qian W. (2021). Treatment-related adverse events of chimeric antigen receptor T-cell (CAR T) in clinical trials: A systematic review and meta-analysis. Cancers.

[25] Atrash S., Moyo T.K. (2021). A Review of Chimeric Antigen Receptor T-Cell Therapy for Myeloma and Lymphoma. Onco Targets.

[26] Neelapu S.S., Tummala S., Kebriaei P., Wierda W., Gutierrez C., Locke F.L., Komanduri K.V., Lin Y., Jain N., Daver N. (2018). Chimeric antigen receptor T-cell therapy—Assessment and management of toxicities. Nat. Rev. Clin. Oncol..

[27] Brudno J.N., Kochenderfer J.N. (2016). Toxicities of chimeric antigen receptor T cells: Recognition and management. Blood.

[28] Hernandez I., Prasad V., Gellad W.F. (2018). Total Costs of Chimeric Antigen Receptor T-Cell Immunotherapy. JAMA Oncol..

[29] Lin J.K., Lerman B.J., Barnes J.I., Boursiquot B.C., Tan Y.J., Robinson A.Q.L., Davis K.L., Owens D.K., Goldhaber-Fiebert J.D. (2018). Cost Effectiveness of Chimeric Antigen Receptor T-Cell Therapy in Relapsed or Refractory Pediatric B-Cell Acute Lymphoblastic Leukemia. J. Clin. Oncol..

[30] Sarkar R.R., Gloude N.J., Schiff D., Murphy J.D. (2019). Cost-Effectiveness of Chimeric Antigen Receptor T-Cell Therapy in Pediatric Relapsed/Refractory B-Cell Acute Lymphoblastic Leukemia. J. Natl. Cancer Inst..

[31] Whittington M.D., McQueen R.B., Campbell J.D. (2020). Valuing Chimeric Antigen Receptor T-cell Therapy: Current Evidence, Uncertainties, and Payment Implications. J. Clin. Oncol..

[32] Whittington M.D., McQueen R.B., Ollendorf D.A., Kumar V.M., Chapman R.H., Tice J.A., Pearson S.D., Campbell J.D. (2018). Long-term Survival and Value of Chimeric Antigen Receptor T-Cell Therapy for Pediatric Patients With Relapsed or Refractory Leukemia. JAMA Pediatr..

[33] Petrou P. (2019). Is it a Chimera? A systematic review of the economic evaluations of CAR-T cell therapy. Expert Rev. Pharm. Outcomes Res..

[34] Upadhaya S., Yu J.X., Shah M., Correa D., Partridge T., Campbell J. (2021). The clinical pipeline for cancer cell therapies. Nat. Rev. Drug Discov..

[35] Bishop M.R., Dickinson M., Purtill D., Barba P., Santoro A., Hamad N., Kato K., Sureda A., Greil R., Thieblemont C. (2022). Second-line tisagenlecleucel or standard care in aggressive B-cell lymphoma. N. Engl. J. Med..

[36] Locke F.L., Miklos D.B., Jacobson C.A., Perales M.A., Kersten M.J., Oluwole O.O., Ghobadi A., Rapoport A.P., McGuirk J., Pagel J.M. (2022). Axicabtagene ciloleucel as second-line therapy for large B-cell lymphoma. N. Engl. J. Med..

